# Diagnostic Value of CA-125 and Other Tumor Biomarkers in Children with Suspected Malignancy: A Retrospective Cohort Study †

**DOI:** 10.3390/diagnostics16010054

**Published:** 2025-12-23

**Authors:** Şule Çalışkan Kamış, Metin Çil, Begül Yağcı

**Affiliations:** Department of Pediatric Hematology and Oncology, Adana City Education and Research Hospital, Adana Faculty of Medicine, University of Health Sciences, 01370 Adana, Turkey

**Keywords:** CA-125, pediatric oncology, tumor markers, ROC analysis, malignancy, CA 19-9, CEA

## Abstract

**Background/Objective**: Tumor biomarkers are widely used in adult oncology, but their diagnostic value in pediatric patients remains unclear. This study aimed to evaluate the clinical significance of CA-125, CA 19-9, CA 15-3, and CEA in children evaluated for suspected malignancy. **Methods**: This retrospective study included 211 pediatric patients (0–18 years) referred to a tertiary pediatric oncology center. Serum levels of the four biomarkers were analyzed in relation to patient demographics, imaging findings, and final diagnoses. Statistical analyses included chi-square, Mann–Whitney U, Kolmogorov–Smirnov tests, and receiver operating characteristic (ROC) curve analysis. **Results**: Of the 211 patients, 35 (16.6%) were diagnosed with malignancy. Elevated CA-125 was significantly associated with malignancy (*p* = 0.002), particularly among postpubertal female patients. Imaging abnormalities were more frequent in CA-125–positive cases (*p* < 0.001) and in patients with confirmed malignancy. ROC curve analysis demonstrated that CA-125 had an area under the curve (AUC) of 0.642, indicating modest discriminatory power. No significant associations were found for CA 19-9, CA 15-3, or CEA. **Conclusions**: CA-125 may serve as an adjunctive diagnostic biomarker in pediatric oncology, particularly in postpubertal girls and when interpreted alongside imaging findings. Other markers showed limited diagnostic value. Given its low sensitivity, CA-125 is not suitable as a standalone screening test in children. Larger, multicenter prospective studies are needed to establish pediatric-specific reference ranges and validate these findings.

## 1. Introduction

Tumor biomarkers are substances produced by tumor cells and/or released as part of the host response to cancer, and they are widely used in adult oncology for diagnostic, prognostic, and treatment-monitoring purposes [1,2]. Commonly used markers in adults, such as cancer antigen 125 (CA-125), cancer antigen 19-9 (CA 19-9), cancer antigen 15-3 (CA 15-3), and carcinoembryonic antigen (CEA), play established roles in the detection and follow-up of malignancies including ovarian, colorectal, pancreatic, and breast cancers [1,3].

Childhood cancers account for approximately 1% of all malignancies worldwide, with leukemia, brain tumors, and lymphomas being the most frequently diagnosed entities [4,5]. In pediatric oncology, tumor biomarkers such as alpha-fetoprotein (AFP), beta-human chorionic gonadotropin (β-HCG), neuron-specific enolase (NSE), and urinary catecholamines are more frequently utilized because of their relative specificity for pediatric tumor types, including germ cell tumors and neuroblastoma [4,6]. In contrast, evidence supporting the diagnostic utility of adult-associated tumor biomarkers in children remains limited, and validated pediatric-specific reference intervals are largely lacking [6,7].

CA-125 is best known for its association with epithelial ovarian cancer but may also be elevated in various malignant and benign conditions, including gastrointestinal malignancies [8,9], endometriosis, pelvic inflammatory disease, and ovarian cysts [9,10]. In children, physiological factors such as puberty, hormonal fluctuations, and inflammatory responses can influence CA-125 levels [6,11], complicating interpretation and potentially reducing specificity. Elevations due to benign conditions may lead to false positives, unnecessary imaging, and invasive diagnostic procedures. Limited pediatric studies have reported conflicting results regarding its discriminative power between benign and malignant disease [10,11].

Similarly, CA 19-9, CA 15-3, and CEA are established biomarkers in adult oncology [1,3], yet pediatric data are scarce and often inconclusive [6,7]. Differences in tumor biology, tumor burden, and biomarker expression between children and adults, combined with the reliance on adult-derived reference ranges, may contribute to misclassification and limit clinical utility in the pediatric setting [6,7].

In light of these gaps, the present study aimed to evaluate serum levels of CA-125, CA 19-9, CA 15-3, and CEA in pediatric patients evaluated for suspected malignancy, to determine the frequency of biomarker elevation and to assess their diagnostic performance. Particular emphasis was placed on CA-125, given its reported association with both gynecologic and non-gynecologic tumors and the ongoing uncertainty regarding its clinical relevance in pediatric oncology.

## 2. Materials and Methods

### 2.1. Study Design and Population

This retrospective cohort study was conducted at Adana City Training and Research Hospital, a tertiary referral center for pediatric oncology. We reviewed the records of pediatric patients aged 0–18 years who underwent tumor biomarker testing (CA-125, CA 19-9, CA 15-3, and CEA) between 1 November 2022 and 1 November 2023.

### 2.2. Inclusion and Exclusion Criteria

Inclusion criteria consisted of the availability of tumor biomarker results and complete clinical and imaging data. Patients with known inflammatory, autoimmune, or chronic benign diseases were excluded from the analysis. Patients with missing biomarker values were also excluded to ensure data completeness and analytical accuracy. Based on clinical and histopathological records, patients were classified into two groups: those with confirmed malignancy and those without malignancy.

To address developmental and physiological differences, patients were stratified into prepubertal (<11 years) and postpubertal (≥11 years) groups. The prepubertal cutoff was defined according to established epidemiological data indicating that the average onset of puberty occurs at approximately 11 years in girls and slightly later in boys, and this classification was verified by reviewing documented pubertal findings in the medical records [12].

### 2.3. Biomarker Measurement

Tumor biomarker measurements were performed as part of routine clinical care using an electrochemiluminescence immunoassay (ECLIA) method. In the absence of validated pediatric-specific cut-off values, adult reference ranges were applied (CA-125 > 35 U/mL, CA 19-9 > 37 U/mL, CEA > 5 ng/mL, and CA 15-3 > 30 U/mL). These cut-off values were derived from widely accepted international guidelines and the manufacturer’s assay package inserts [13,14]. Ordering of individual tumor biomarkers was left to the discretion of the treating pediatric hematologist–oncologist according to the patient’s clinical presentation and suspected diagnosis. Consequently, not all patients underwent testing for all four biomarkers, which accounts for the different denominators reported for each biomarker in Table 1.

### 2.4. Ethical Considerations

This study was approved by the Adana City Training and Research Hospital Clinical Research Ethics Committee (Decision No. 2922, dated 9 November 2023). All procedures were conducted in accordance with the principles of the Declaration of Helsinki and institutional ethical standards. Written informed consent was obtained from the parents or legal guardians of all participants. All patient data were anonymized prior to analysis to ensure confidentiality.

### 2.5. Statistical Analysis

All statistical evaluations were performed using IBM SPSS Statistics software version 26.0 (Armonk, NY, USA). Categorical variables are summarized as frequencies and percentages, whereas continuous variables are expressed as mean ± standard deviation or median with interquartile range, depending on the distribution. The normality of continuous variables was assessed using the Kolmogorov–Smirnov test.

For comparisons between two independent groups, Student’s *t*-test was used for normally distributed variables, and the Mann–Whitney U test was applied when normality assumptions were not met. Associations between categorical variables were primarily assessed using the Pearson chi-square test; however, when the assumption of minimum expected cell count (≥5) was violated, the Fisher–Freeman–Halton exact test was employed. Additionally, age-stratified subgroup analyses were conducted using three predefined pediatric age categories (0–1, 1–11, and 11–18 years).

To determine the discriminatory performance of CA-125 in distinguishing malignancy from the non-tumor group, a receiver operating characteristic (ROC) curve was generated. The optimal cut-off value was calculated using the Youden index, and sensitivity, specificity, positive predictive value (PPV), negative predictive value (NPV), and area under the ROC curve (AUC) were reported with 95% confidence intervals.

Multivariable logistic regression analysis was performed to evaluate the independent predictive contribution of CA-125 levels, demographic variables (age and sex), and radiological abnormalities to malignancy risk. Correlations between continuous variables were assessed using Pearson’s correlation coefficient for normally distributed data and Spearman’s rank correlation for non-normally distributed data.

All statistical tests were two-tailed, and a *p*-value of ≤0.05 was considered statistically significant.

## 3. Results

A total of 211 pediatric patients aged 0–18 years who underwent tumor biomarker testing (CA-125, CA 19-9, CA 15-3, and CEA) at Adana City Training and Research Hospital between 1 November 2022 and 1 November 2023 were retrospectively included in this study. Among them, 145 (68.7%) were female, and 66 (31.3%) were male, with a median age of 13 years (range: 0–17 years). In the postpubertal female subgroup (*n* = 107), 13 malignancy cases were identified.

### 3.1. Patient Selection Criteria

Inclusion criteria included pediatric patients (0–18 years) who had at least one tumor biomarker tested (CA-125, CA 19-9, CA 15-3, or CEA) during the study period. Exclusion criteria included incomplete medical records, previously diagnosed malignancies already receiving treatment at the time of biomarker testing, and patients lost to follow-up. Ethical approval was obtained, and patient confidentiality was maintained through anonymized data collection.

### 3.2. Patient Stratification and Study Groups

Based on final diagnoses confirmed through pathology and/or imaging, patients were classified into two main groups:

- Oncologic tumor group: 35 patients (16.6%) with confirmed oncologic diagnoses, including malignant tumors as well as selected benign or low-grade lesions that were managed and followed by the pediatric oncology team.

This group included entities such as mature cystic teratoma, mesoblastic nephroma, hepatic tumors, and adrenocortical adenoma, which may exhibit benign or low-grade biological behavior but required oncologic evaluation due to their clinical and radiological features.

- Non-tumor group: 176 patients (83.4%) with benign conditions such as cysts, reactive lymphadenopathy, or functional imaging findings.

To assess age-related biological variation, patients were further stratified into the following groups:

- Prepubertal group (<11 years): 72 patients;
- Postpubertal group (≥11 years): 139 patients.

In addition to puberty-based grouping, patients were also categorized into three pediatric age ranges (0–1, 1–11, and 11–18 years) to account for potential developmental differences, particularly those associated with infancy.

### 3.3. Tumor Biomarker Testing and Positivity

Tumor biomarkers were ordered as part of routine clinical practice. The frequency of testing and elevated results, based on standard adult reference values, are presented in Table 1.

Of the 27 patients with elevated CA-125, 8 (29.6%) were diagnosed with malignancies. Chi-square analysis revealed a significant association between elevated CA-125 and malignancy (*p* = 0.002). No statistically significant associations were observed for CA 19-9 (*p* = 0.533, chi-square test), CEA (*p* = 0.573, chi-square test), or CA 15-3 (*p* = 0.711, Fisher’s exact test).

### 3.4. Distribution of Malignancy Diagnoses

Among the 35 patients in the oncologic tumor group, the most frequent diagnoses were acute lymphoblastic leukemia (14.3%), non-Hodgkin lymphoma (11.5%), neuroblastoma (11.5%), and ovarian tumors (11.5%). Less common tumors in this group included mature cystic teratoma, Ewing sarcoma, pontine glioma, and other rare pediatric neoplasms (Table 2).

This group comprises malignant tumors as well as selected benign or low-grade lesions (e.g., mature cystic teratoma, mesoblastic nephroma, hepatic tumor, adrenocortical adenoma) that were referred to and managed by the pediatric oncology team.

### 3.5. Subgroup Analysis by Pubertal Status

Among the postpubertal group (*n* = 139), CA-125 was elevated in 24 patients (17.3%), with 7 malignancy cases. In contrast, only 3 prepubertal patients had elevated CA-125, of whom one had a malignancy. The difference in elevation rates between the two groups was statistically significant (*p* = 0.041, chi-square test).

### 3.6. Sex-Based Analysis

CA-125 positivity was more prevalent in females (22 out of 27 cases, 81.5%). However, there was no statistically significant association between sex and malignancy in relation to tumor marker levels (*p* > 0.05, chi-square test).

### 3.7. Radiological Findings

Imaging studies were performed in 187 patients (88.6%), with abnormal findings in 148 (79.1%). Among these, the following observations were made:

- Cystic lesions: 87 patients (58.8%);
- Solid masses: 29 patients (19.6%);
- Lymphadenopathy: 22 patients (14.9%).

Abnormal imaging was significantly more frequent in the oncologic tumor group compared to the non-tumor group (94.3% vs. 75.0%; *p* = 0.018, chi-square test).

### 3.8. Graphical Representation of Key Findings

**Figure 1:**
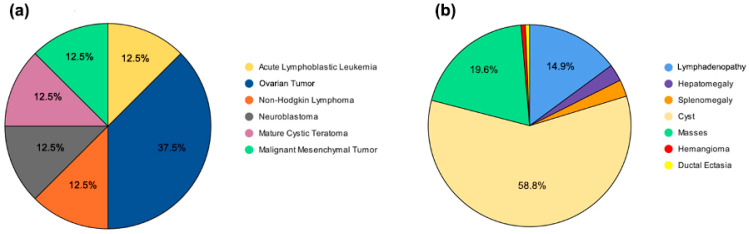
(**a**) Malignancies detected in patients with elevated CA-125 levels. (**b**) Radiological findings in 148 patients with abnormal imaging results. The most common finding was cystic lesions (58.8%), followed by solid masses (19.6%), lymphadenopathy (14.9%); other findings were observed less frequently.

Visual summaries of CA-125-positive malignancies and imaging findings are provided in Figure 1a,b, respectively. Figure 1a shows that the most frequent malignancy among patients with elevated CA-125 was ovarian tumors (37.5%), followed by acute lymphoblastic leukemia (12.5%), non-Hodgkin lymphoma (12.5%), neuroblastoma (12.5%), mature cystic teratoma (12.5%), and malignant mesenchymal tumor (12.5%).

### 3.9. Serial Biomarker Monitoring

Serial tumor marker measurements were available for 20 patients. Biomarker levels decreased in 13 patients (65%), increased in 6 patients (30%), and remained stable in one case (5%). These trends correlated with clinical outcomes and imaging follow-up, although timing was variable due to the retrospective nature of the study.

### 3.10. Diagnostic Performance of CA-125

The sensitivity, specificity, PPV, and NPV of CA-125 for detecting malignancy were 22.9%, 85.2%, 29.6%, and 80.4%, respectively. The area under the ROC curve (AUC) was 0.642 (95% CI: 0.466–0.819, *p* = 0.066), indicating limited discriminative ability.

**Figure 2:**
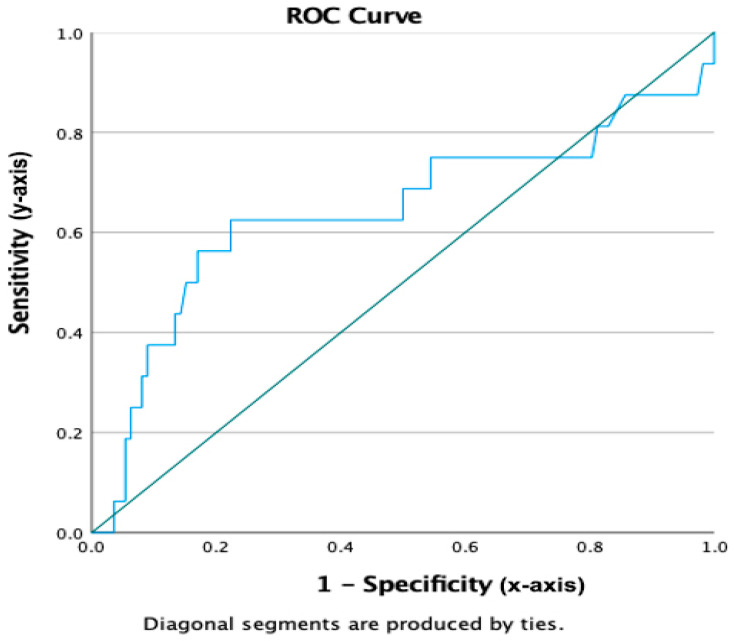
ROC curve for CA-125 in detecting malignancy. Axes are labeled as ‘Sensitivity’ (*y*-axis) and ‘1—Specificity’ (*x*-axis). The area under the curve (AUC) was 0.642 (95% CI: 0.466–0.819, *p* = 0.066). The optimal cut-off value determined by the Youden index was 30.2 U/mL, yielding a sensitivity of 62.5% and a specificity of 77.7% for detecting malignancy.

The optimal cut-off value determined by the Youden index was 30.2 U/mL, which yielded a sensitivity of 62.5% and a specificity of 77.7% for detecting malignancy. These findings suggest that CA-125 may serve as an adjunctive diagnostic marker in pediatric oncology when interpreted alongside clinical and radiological findings (Table 3, Figure 2).

Age-stratified analysis using the reviewer-recommended categories (0–1, 1–11, and 11–18 years) did not demonstrate a significant association between age group and elevated CA-125 levels. Because more than 50% of the cells had expected counts below 5, Pearson’s chi-square test was not appropriate; therefore, the Fisher–Freeman–Halton exact test was used. The association was not statistically significant (*p* = 1.000).

## 4. Discussion

This study evaluated the diagnostic utility of multiple tumor biomarkers—particularly CA-125—in identifying malignancies among pediatric patients. Elevated CA-125 levels were significantly associated with malignancy, especially among postpubertal females, suggesting potential clinical value as a supportive diagnostic indicator when interpreted in conjunction with imaging and clinical findings. However, the biomarker demonstrated limited sensitivity but acceptable specificity, consistent with a “rule-in” rather than “rule-out” diagnostic profile. Therefore, CA-125 should not be used as a screening tool, as its low sensitivity at standard adult cut-offs (22.9%) would miss a substantial proportion of malignancies. Although age 11 years was used as a proxy for pubertal onset, this approach does not fully reflect physiological pubertal development; Tanner staging, which was unavailable in this retrospective dataset, would provide a more accurate assessment. This limitation may influence the interpretation of age-related biomarker variation.

Compared with adults, pediatric data on tumor biomarkers are scarce. While CA-125 is classically associated with epithelial ovarian cancer [15], it can also be elevated in various benign and inflammatory conditions such as endometriosis, pelvic inflammatory disease, functional ovarian cysts, and menstruation-related peritoneal irritation [16]. In children, additional benign causes—including intra-abdominal inflammation and peritoneal irritation—may further contribute to false-positive results. Therefore, CA-125 should be viewed strictly as an adjunctive marker in pediatric clinical practice, particularly in adolescent girls.

Despite these limitations, our findings support a potential role for CA-125 within a multimodal diagnostic framework integrating clinical, laboratory, and radiological assessments. Notably, 29.6% of patients with elevated CA-125 had a malignancy, consistent with previous studies reporting modest predictive performance [17,18]. By incorporating pubertal stratification, our study expands existing literature and suggests that hormonal or developmental factors may modulate CA-125 expression in pediatric patients [12,17]. Similar to AFP, several pediatric tumor biomarkers exhibit physiological age-related variation. To explore potential developmental differences in biomarker expression, we performed an additional age-stratified analysis using three pediatric categories (0–1, 1–11, and 11–18 years). Although the 0–1 year group included only one patient, this category was retained to reflect the distinct physiological characteristics of early infancy.

In contrast, we found no significant associations between malignancy and the other biomarkers (CA 19-9, CA 15-3, CEA). Several factors may explain these findings, including limited sample size, lower tumor burden in children, nonspecific biomarker elevation in benign conditions, and fundamental differences in tumor biology between pediatric and adult populations [18,19,20]. Interpretation is further complicated by the absence of pediatric-specific reference intervals; the reliance on adult cut-off values may underestimate abnormal biomarker expression in children and thereby reduce diagnostic sensitivity [6,18]. Collectively, these findings highlight the need for pediatric-tailored biomarker research.

Tumor-specific biomarkers such as AFP, β-HCG, urinary catecholamines, ferritin, and LDH remain the primary diagnostic tools in pediatric oncology because they provide tumor-type–specific information that directly guides clinical evaluation. In this retrospective cohort, however, these biomarkers were obtained selectively rather than uniformly across all 211 patients; therefore, a formal head-to-head comparison of diagnostic performance between CA-125 and these established pediatric markers was not feasible. In routine clinical practice at our center, CA-125 functions solely as an adjunctive parameter and is not intended to replace these standard tumor markers.

Radiological abnormalities were strongly associated with malignancy and enhanced diagnostic yield when combined with CA-125 elevation. This supports the integration of serum biomarkers with imaging findings to guide decisions regarding advanced imaging or biopsy [10,18]. Additionally, CA-125 elevation in benign lesions such as mature cystic teratomas, as observed here and in previous studies [16], highlights the necessity of interpreting results within the broader clinical and developmental context.

With regard to biomarker mechanisms, CA-125 is known to be expressed by mesothelial cells lining the peritoneum, pleura, and reproductive organs. Its elevation in non-gynecologic pediatric tumors—such as lymphomas, neuroblastoma, or mesenchymal tumors—may reflect tumor-related peritoneal irritation, cytokine-mediated serosal activation, or secondary inflammatory responses. Further mechanistic studies are required to clarify these pathways.

Adult data on CA-125 are largely derived from studies on ovarian tumors and adnexal masses [19,20]. While ovarian tumors were present in our cohort, differences in tumor biology and patient age limit the direct applicability of adult findings to the pediatric population.

### 4.1. ROC Analysis

The ROC curve analysis revealed an AUC of 0.642 (95% CI: 0.466–0.819), indicating only fair discriminative capacity. While markedly lower than values reported for adult ovarian cancer (often >0.85), the AUC is consistent with prior pediatric or mixed-age studies [17,18]. The optimal cut-off identified using the Youden index (30.20 U/mL) provided improved sensitivity (62.5%) while maintaining acceptable specificity (77.7%), suggesting that pediatric cohort-specific thresholds may enhance diagnostic performance. Even with modest AUC values, CA-125 may retain clinical usefulness as part of an integrated diagnostic model rather than a stand-alone test.

Future research should prioritize multicenter prospective studies including healthy controls, enabling the development of pediatric-specific cut-off values. Emerging biomarkers such as HE4, AFP-L3, and circulating tumor DNA (ctDNA), as well as molecular profiling tools including proteomics and metabolomics, warrant further evaluation for their potential diagnostic and prognostic roles in pediatric oncology [20,21]. Characterizing the underlying biological mechanisms regulating biomarker expression may further enhance early detection strategies and personalized diagnostic algorithms [21,22].

### 4.2. Limitations

This study has several limitations. First, it is a retrospective analysis conducted at a single tertiary care center, which may limit the generalizability of the findings. Second, the one-year study period may not adequately capture long-term trends in biomarker fluctuations or clinical outcomes. Third, because validated pediatric-specific cutoff values are lacking, adult reference ranges were used to define biomarker positivity, which may reduce diagnostic sensitivity in children. Additionally, baseline tumor marker measurements are not obtained in healthy children at our institution due to ethical and practical constraints, preventing the establishment of institution-specific pediatric reference intervals.

Although age 11 years was used as a proxy for pubertal onset, this approach does not fully reflect clinical pubertal development. Tanner staging would provide a more accurate assessment; however, Tanner data were not available in this retrospective dataset, limiting the precision of age-related biomarker interpretation. Furthermore, some subgroup analyses—particularly those involving postpubertal females with malignancy—were based on small sample sizes, which may limit statistical power and the robustness of these findings.

Finally, the absence of a healthy control group restricts the ability to determine the specificity of biomarker elevations in distinguishing benign from malignant conditions. The potential for false-positive CA-125 elevations in common pediatric benign conditions (e.g., functional ovarian cysts, menstruation-related changes, peritoneal or intra-abdominal inflammation) must also be acknowledged.

## 5. Conclusions

This study suggests that CA-125 may have a limited adjunctive role in the diagnostic evaluation of pediatric patients with suspected malignancy, particularly among postpubertal children and when interpreted in conjunction with radiological findings. Although elevated CA-125 levels were significantly associated with malignancy, the biomarker demonstrated modest discriminatory performance on ROC curve analysis (AUC: 0.642) and limited sensitivity, underscoring that it should not be used as a standalone diagnostic or screening tool in pediatric populations. Other biomarkers, including CA 19-9, CA 15-3, and CEA, did not show significant diagnostic associations in this cohort.

Importantly, the findings of this study should be regarded as exploratory and interpreted with caution due to the retrospective design, single-center setting, limited sample size for subgroup analyses, and reliance on adult-derived reference ranges. In addition, physiological factors such as pubertal development may influence biomarker levels and further complicate clinical interpretation in children and adolescents.

Accordingly, the clinical integration of less specific tumor biomarkers such as CA-125 in pediatric oncology remains uncertain, and their use should be restricted to a multimodal diagnostic framework that incorporates clinical assessment, imaging findings, and established pediatric tumor-specific biomarkers. Larger, multicenter prospective studies—including healthy pediatric control populations—are required to establish age- and puberty-adjusted reference values and to clarify the appropriate clinical role of such nonspecific biomarkers. Preliminary results of this cohort were previously presented as a conference abstract [23]; the present manuscript provides a comprehensive and extended analysis of these findings.

## Figures and Tables

**Table 1:** Frequency of tumor marker testing and positivity.

Biomarker	Cut-Off	Patients Tested (*n*)	Elevated Results (*n*, %)
CA-125	>35 U/mL	128	27 (21.1%)
CA 19-9	>37 U/mL	122	11 (9.0%)
CEA	>5 ng/mL	187	9 (4.8%)
CA 15-3	>30 U/mL	117	1 (0.9%)

**Table 2:** Diagnoses of patients in the oncologic tumor group (*n* = 35).

Diagnoses	Number (*n*)	%
Acute Lymphoblastic Leukemia (ALL)	5	14.3
Non-Hodgkin Lymphoma (NHL)	4	11.5
Neuroblastoma (NBL)	4	11.5
Ovarian Tumor	4	11.5
Mature cystic teratoma (MCT)	3	8.6
Ewing Sarcoma (EWS)	2	5.9
Pontine glioma	2	5.9
Acute Myeloid Leukemia (AML)	1	2.8
Osteosarcoma (OST)	1	2.8
Rhabdomyosarcoma (RMS)	1	2.8
Langerhans Cell Histiocytosis (LCH)	1	2.8
Pilocytic Astrocytoma	1	2.8
Malignant Mesenchymal Tumor	1	2.8
Nasopharyngeal Cancer	1	2.8
Adrenocortical Adenoma	1	2.8
Mesoblastic Nephroma	1	2.8
Yolk Sac Tumor	1	2.8
Hepatic Tumor	1	2.8
Total	35	100

**Table 3:** Diagnostic performance measures for CA-125 in detecting malignancy.

Metric	Value (%)	95% CI
Sensitivity	22.9	10.4–40.1
Specificity	85.2	78.6–90.6
Positive Predictive Value (PPV)	29.6	13.8–50.2
Negative Predictive Value (NPV)	80.4	73.0–86.5
Area Under Curve (AUC)	0.64	0.54–0.74

Abbreviations: PPV, positive predictive value; NPV, negative predictive value; AUC, area under the curve.

## Data Availability

Data supporting the findings of this study are available upon reasonable request from the corresponding author.
